# Genome-Wide Identification, Characterization and Expression Analysis of the Chalcone Synthase Family in Maize

**DOI:** 10.3390/ijms17020161

**Published:** 2016-01-27

**Authors:** Yahui Han, Ting Ding, Bo Su, Haiyang Jiang

**Affiliations:** Key Laboratory of Crop Biology of Anhui Province, Anhui Agricultural University, Hefei 230036, China; hyahui@163.com (Y.H.); dingting98@126.com (T.D.); subo315@163.com (B.S.)

**Keywords:** chalcone synthase, genome-wide analysis, expression, evolution, maize

## Abstract

Members of the chalcone synthase (CHS) family participate in the synthesis of a series of secondary metabolites in plants, fungi and bacteria. The metabolites play important roles in protecting land plants against various environmental stresses during the evolutionary process. Our research was conducted on comprehensive investigation of *CHS* genes in maize (*Zea mays* L.), including their phylogenetic relationships, gene structures, chromosomal locations and expression analysis. Fourteen *CHS* genes (*ZmCHS01–14*) were identified in the genome of maize, representing one of the largest numbers of *CHS* family members identified in one organism to date. The gene family was classified into four major classes (classes I–IV) based on their phylogenetic relationships. Most of them contained two exons and one intron. The 14 genes were unevenly located on six chromosomes. Two segmental duplication events were identified, which might contribute to the expansion of the maize *CHS* gene family to some extent. In addition, quantitative real-time PCR and microarray data analyses suggested that *ZmCHS* genes exhibited various expression patterns, indicating functional diversification of the *ZmCHS* genes. Our results will contribute to future studies of the complexity of the *CHS* gene family in maize and provide valuable information for the systematic analysis of the functions of the *CHS* gene family.

## 1. Introduction

Chalcone synthase (CHS, enzyme commission number: E.C. 2.3.1.74) is an important enzyme which catalyzes the first key step during the progress of flavonoid biosynthesis. CHS condenses three acetate units of malonyl-coenzyme A (malonyl-CoA) with a phenylpropanoid CoA ester (*p*-coumaroyl-CoA) to generate a chalcone, which is the precursor of various flavonoids [1]. Recent studies about the *CHS* gene family indicated that generation and loss of genes frequently occurred, and that *CHS* and non-*CHS* genes had co-evolved throughout the evolutionary process of angiosperms [2]. The enzyme family is also known as type III polyketide synthases [3], which exist in all the plant species. Members of the CHS family are homodimers of 40–45 kDa subunits, and possess a Cys-His-Asn catalytic triad (CHN) in their active sites [2]. They show similarity in sequences, structures and catalytic principles [3,4]. CHS is a representative of the CHS family. This family also includes other members such as biphenyl synthase [5], 2-pyrone synthase (2-PS) [6], benzalacetone synthase [7], stilbene synthase [8], bibenzyl synthase [9], benzophenone synthase [10], pentaketide chromone synthase [11], octaketide synthase [12] and *p*-coumaroyltriacetic acid synthase [13]. All these other enzymes have differences from CHS in many aspects, such as the choice of initial substrates, the number of condensation reactions, and the mechanisms by which the resulting intermediates are cyclized and aromatized. Stilbene synthase (STS), which was identified in pines and peanuts, exhibits an identity of >65% with CHS protein sequences and also condenses three acetate units of malonyl-CoA with a phenylpropanoid CoA ester. Unlike CHS, STS cyclizes the intermediate by another pathway, in which CO_2_ is lost to generate resveratrol, a phytoalexin stilbene. By contrast, 2-PS in gerbera condenses acetyl-CoA with two malonyl-CoA to generate a pyrone, which is converted into antifeedant glucoside metabolites [14]. Therefore, the CHS family together participates in the biosyntheses of various natural products, which play significant roles in flower pigmentation, pollen fertility and protection against UV. *CHS* genes have been widely studied because of their important functions. A number of aspects on regulation and function of *CHS* family have been investigated in many plants, including grape [15], *Gerbera hybrida* [16], apple [17], *Oncidium* orchid [18], *Hypericum sampsonii* [10], Arabidopsis [19], Petunia [20] and bean [21]. Two maize *CHS* genes were previously reported, *C2* and *Whp* [22,23]. Moreover, one anther specific chalcone synthase-like (*ASCL*) gene (*ZmCHSL*, NP_001149508) was also reported by Jepson *et al.* [24]. Products of 15 or even more genes are essential for anthocyanin biosynthesis in maize [25], including *CHS* gene *C2*, which probably have a bearing on the production of the anthocyanin pigment [26,27]. The *CHS* family had also been studied comprehensively in *Physcomitrella patens* [28].

Recently, the whole genome assembly of maize (*Zea mays* L. B73) has been completed [29], which made it possible to perform a genome-wide investigation of the *CHS* family to analyze their evolutionary processes and functional diversification in this species. In our study, we carried out an overall analysis of the *CHS* family by searching the whole genome of maize. Furthermore, many studies reported that the expression of *CHS* genes in different plants was induced by a number of biotic and abiotic stress responses [30]. In this study, *ZmCHS* transcripts were measured and showed enhanced expression after salicylic acid (SA) treatment. Results of this study will provide a new start for the future studies of the functional diversification and evolutionary process of the *CHS* family in angiosperms.

## 2. Results

### 2.1. Identification and Annotation of Chalcone Synthase (CHS) Genes in Maize

Sixty-five candidate CHS protein sequences were identified by querying the maize genome database with the consensus protein sequences of the CHS family. Whereafter, all candidate CHS proteins were investigated for the presence of the Chal_sti_synt_C (PF02797) and Chal_sti_synt_N (PF00195) domains by searching Pfam Datebase [31]. The two domains were predicted to possess molecular function of transferase activity and transferring acyl groups (GO:0016746) according to Gene Ontology [32]. In addition, Chal_sti_synt_N domain possess molecular function of biological process biosynthetic process (GO:0009058). Finally, fifty-one protein sequences were discarded because they lacked the two domains or represented overlapping genes, and 14 non-redundant *CHS* genes (named *ZmCHS01–14*) were identified and described (Table 1).

**Table 1:** The chalcone synthase family genes of maize.

Number	Gene Name	Translation Product	*M*w (Da)	Size (aa)	pI	Chromosome
1	*ZmCHS01*	GRMZM2G422750_P03	43195.59	400	6.33	4
2	*ZmCHS02*	GRMZM2G151227_P01	43325.61	401	6.02	2
3	*ZmCHS03*	GRMZM2G175812_P01	43006.2	402	5.74	7
4	*ZmCHS04*	AC191551.3_FGP003	43065.52	396	5.98	3
6	*ZmCHS05*	GRMZM2G435393_P01	43425.83	398	6.28	3
7	*ZmCHS06*	GRMZM2G346095_P01	42391.54	397	6.19	5
8	*ZmCHS07*	GRMZM2G009348_P01	49692.84	472	8.9	4
9	*ZmCHS08*	GRMZM2G009510_P01	44829.17	420	6.08	4
10	*ZmCHS09*	GRMZM2G027130_P01	44136.6	420	5.83	2
11	*ZmCHS10*	GRMZM2G114471_P01	43556.78	405	5.46	4
12	*ZmCHS11*	GRMZM2G108894_P01	45994.51	427	5.35	7
13	*ZmCHS12*	GRMZM2G131529_P01	35275.5	326	5.77	3
14	*ZmCHS13*	GRMZM2G477683_P01	45574.27	421	8.08	5
15	*ZmCHS14*	GRMZM2G380650_P02	42110.41	390	6.92	1

*M*w: molecular mass; pI: isoelectric point.

### 2.2. Phylogenetic and Structural Analysis of the Putative Maize CHS Proteins

**Figure 1:**
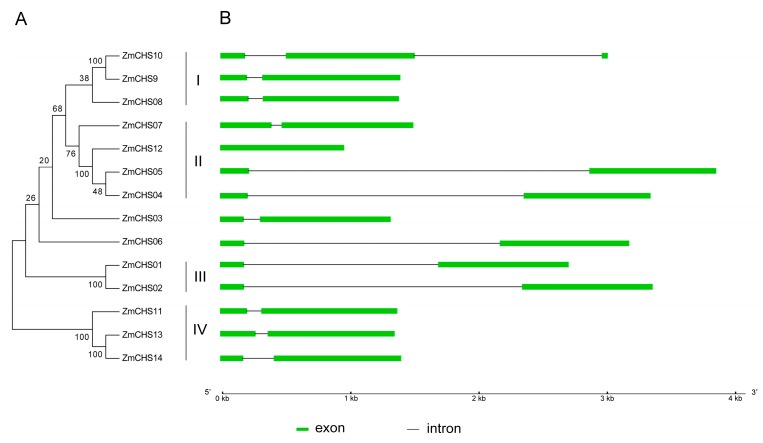
Phylogenetic tree and gene structure of the 14 predicted maize chalcone synthases (*CHS*s). (**A**) The unrooted neighbor-joining tree was constructed using the MEGA4.0 program. The bootstrap values, which were produced using 1000 replicates with the pairwise deletion option, are noted at each node; (**B**) Exon-intron structure was generated using Gene Structure Display Server (GSDS). The exons and introns are indicated by green boxes and gray lines, respectively. The scale at the bottom is in kilobases.

An unrooted phylogenetic tree (Figure 1) was generated by multiple sequence alignment of the protein sequences of all the *ZmCHS* genes. The *ZmCHS* genes were classified into four major groups (classes I, II, III and IV) based on the phylogenetic tree, with supported bootstrap values. Subsequently, gene structure (including exons and introns) analysis was done to support the phylogenetic analysis. Additional sequence alignment analysis was conducted to reveal the genetic relationship between the 14 *ZmCHS*s and *C2*, *Whp* and *ZmCHSL* [22,23,24]. The three gene pairs (*ZmCHS01-C2*, *ZmCHS02*-*Whp*, *ZmCHS11-ZmCHSL*) showed high identities of 100%, 96.76% and 97.66% respectively in their sequence alignment. Therefore, we confirmed that *ZmCHS01* is *C2*, and *ZmCHS02* and *ZmCHS11* are likely to be *Whp* and *ZmCHSL*, respectively. The schematic structures revealed that most *ZmCHS* genes exhibited similar gene structures, two exons and one intron. While *ZmCHS12* contained only one exon, and *ZmCHS10* had multiple exons and introns.

### 2.3. Conserved Motifs of the Putative ZmCHS Proteins and Sequence Alignment against Other Plant CHSs

**Figure 2:**
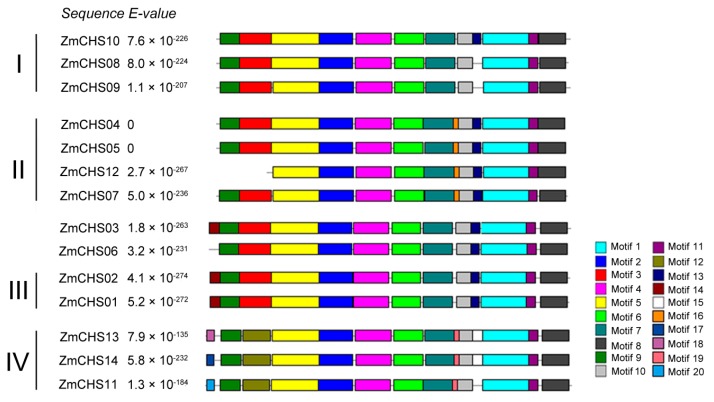
Distribution of 20 conserved motifs in the putative CHS proteins of maize. Motifs in the putative ZmCHS proteins were obtained using the Multiple Em for Motif Elicitation (MEME) web server. The length of each box does not represent the actual size of each motif.

**Figure 3:**
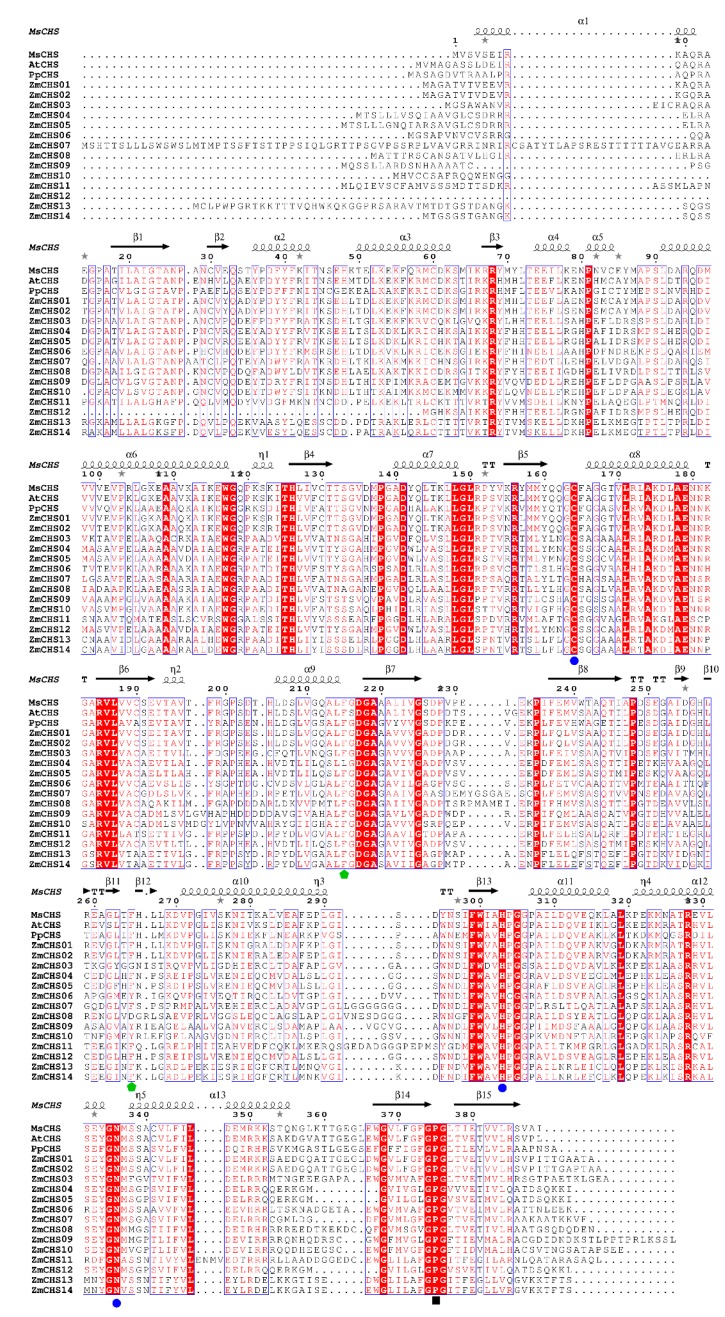
Sequence alignment of ZmCHSs against the other plant CHSs. The first line represents the secondary structure of *Medicago sativa* chalcone synthase (MsCHS). The blue boxes and red letters represent conserved residues. The red regions represent sequences of strict sequence conservation. The black wave lines and black arrows represent α-helix and β-pleated sheet. The catalytic triad of CHSs, residues connected with CoA-binding and CHS family-specific Pro375 are labeled with blue points, green pentagrams and black quadrangles at the bottom of the sequences, respectively. MsCHS = *Medicago sativa* (alfalfa) chalcone synthase (P30074); AtCHS = Arabidopsis chalcone synthase (AAB35812.1); PpCHS = *Physcomitrella patens* chalcone synthase (ABB84527.1).

Twenty conserved motifs were investigated in the putative CHS proteins of maize (Figure 2). The detailed information of the 20 motifs is listed in Table 2 and also shown in Figure 2. We categorized the 14 ZmCHSs into four classes (Figure 2) according to their phylogenetic relationships. The Chal_sti_synt_C domain was represented by motifs 1 and 8. And the Chal_sti_synt_N domain was represented by motifs 2, 3, 4, 5, 6 and 9 (Table 2). The two domains were highly conserved in almost all of the putative ZmCHS proteins, except that ZmCHS12 lacks part of the Chal_sti_synt_N domain. Motifs 4 and 1 contained Cys164 and His303 and Asn336 respectively of the characteristic catalytic triad (Figure 2, Figure 3 and Table 2). The catalytic triad inherited from the ketoacyl synthase III (KAS III) ancestor [3] is also highly conserved in all of the ZmCHSs (Figure 3). In addition, many motifs were conserved within a subfamily. For example, motif 16 was present in all members of class II. All members of class IV contained conserved motifs 12 and 19. The subfamily-specific motifs might be required for subfamily-specific functions. However, some motifs (motif 1, 2, 4, 5, 6, 7, 8, 10, 11) are distributed in every subfamily. So these motifs might be important for the functions of ZmCHS proteins.

**Table 2:** Detailed information of the 20 motifs in the putative maize chalcone synthase (CHS) proteins.

Motif	Width	Best Possible Match	Domain
1	50	WNDLFWAVHPGGPAILDQVEACLKLQPHKLKASRHVLSEYGNMSSPTVIF	Chal_sti_synt_C
2	36	EWGRPATDITHLVFCTYSGAHMPGVDWQLASLLGLR	Chal_sti_synt_N
3	34	NCVYQDEYPDYYFRITKSEHLTDLKEKFKRICHK	Chal_sti_synt_N
4	38	RTMLYMNGCSGGCAALRVAKDMAENNRGARVLVACAEM	Chal_sti_synt_N
5	50	IKKRYFHHTEELLREHPEFIDYSMPSLHERQDIMNSAVPELAAAAAQKAI	Chal_sti_synt_N
6	31	FRPPHEDHPYTLIGQALFGDGAGAVIVGADP	Chal_sti_synt_N
7	31	VERPIFEMVSASQTMIPDSEHVIDGQLCEDG	*
8	28	CEWGVMVGFGPGFTVETMVLHACKKTKK	Chal_sti_synt_C
9	21	RKWQRADGPATVLAIGTANPP	Chal_sti_synt_N
10	15	REIPSLIEENIEQCM	*
11	9	VLDELRRRQ	*
12	29	PQEKVVDSYLQESSCDDPDTRAKLQRLCT	*
13	8	DAFSPLGI	*
14	11	MAGATVTVEEV	*
15	11	RTLMNKVGIKD	*
16	6	LHFNPS	*
17	6	IDQFIN	*
18	6	VQHWKK	*
19	6	INFKLG	*
20	7	QIEYSCF	*

The detailed information of the 20 motifs are shown in the table, including their width, best possible match sequences, and domains they are contained in; *: represents the motifs that are not included in the domain sequences.

The “gatekeeper” phenylalanines connected with CoA-binding at positions 215 and 265 [3] are also conserved. The exceptions to these conservations are ZmCHS04 which contains a lysine substituted for phenylalanine at position 215, and ZmCHS03, -06, -08, -09 and -10 which contain other amino acid residues substituted for phenylalanine at position 265 (Figure 3), which probably led to remarkable functional diversity, such as the choice of the initial substrates. The CHS family-specific Pro375 residues [3] were also maintained in all the ZmCHSs (Figure 3). The secondary structure of the putative maize CHS proteins was analyzed with the secondary structure of *Medicago sativa* chalcone synthase (MsCHS) as a template using online server PDB and ESpript. An alignment of MsCHS, ZmCHSs and other plant CHSs was also performed using the ClustalW software. All these results indicated that ZmCHSs exhibited high similarity in sequences with MsCHSs and other plant CHSs, suggesting that the CHS family is conserved during the evolutionary process (Figure 3).

### 2.4. Chromosomal Location, Gene Duplication

**Figure 4:**
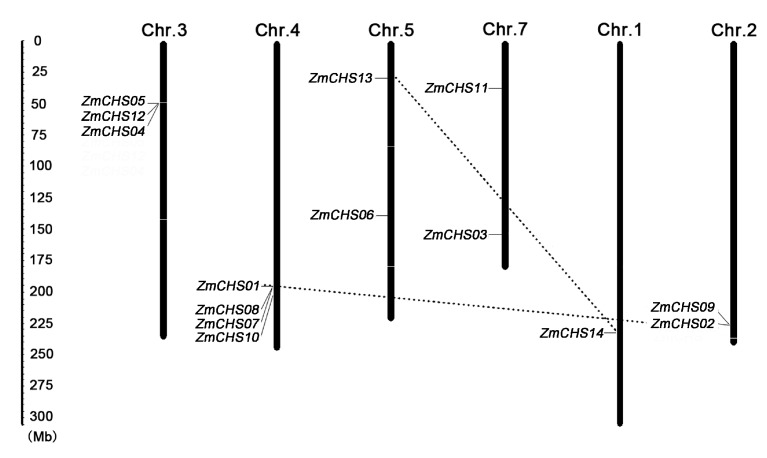
Location of 14 *CHS* genes on maize chromosomes. The “Chr.” at the top of each bar represent the chromosome number of maize. The location of each *CHS* gene is indicated by corresponding gene name at the left of corresponding chromosome. The segmental duplicated genes are connected by dashed lines. The scale on the left is in megabases.

A physical map was drawn to show the distribution of *ZmCHS*s on different chromosomes of maize. The 14 *ZmCHS*s were located unevenly on chromosome number 1, 2, 3, 4, 5 and 7 (Figure 4). Chromosome 4 contained the largest number of *ZmCHS*s (four) followed by chromosome 3 (three), and each of chromosomes 2, 5 and 7 contained two *ZmCHS* genes. Chromosomes 6, 8, 9 and 10 had no *CHS* genes.

Gene duplication events, including tandem and segmental duplications, are considered as a driving force for the expansion of many gene families during the evolutionary process [33,34]. In this study, two gene pairs (*ZmCHS01/02*, *ZmCHS13/14*) were found to be involved in segmental repeats (Figure 4). They shared a similar evolutionary process and were closely related to each other according to their phylogenetic relationships and gene structures (Figure 1). So the two gene pairs were considered as segmental duplications. In contrast, no tandem duplications occurred.

**Figure 5:**
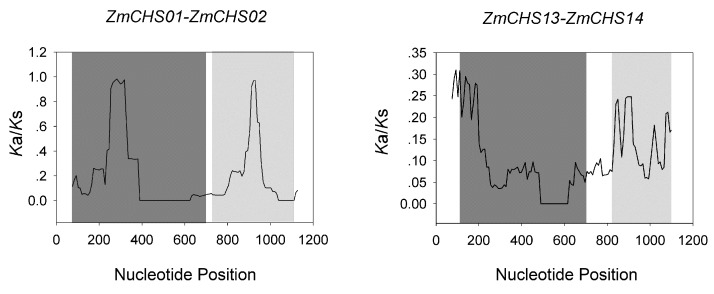
Sliding window plots of duplicated *CHS* genes. The gray blocks, from dark to light, indicate the positions of the Chal_sti_synt_N domain and Chal_sti_synt_C domain, respectively. The window size was 150 bp and the step size was 9 bp.

To explore the selective constraints among the two pairs of duplicated ZmCHS genes, their ratios of the number of nonsynonymous substitutions per non-synonymous site (*K*a) to the number of synonymous substitutions per synonymous site (*K*s) (*K*a/*K*s) were calculated (Table 3). In general, a *K*a/*K*s ratio of >1 represents positive selection with promoted evolution; a ratio of 1 represents neutral selection and a ratio of <1 represents negative or purifying selection. In our study, *K*a/*K*s ratios of the two pairs of duplicated *CHS* genes were <1, indicating that after the gene duplication events, these duplicated *ZmCHS* genes evolved under purifying selection with narrow functional divergence (Table 3, Figure 5). Because some positive selection might be shielded by strong purifying selection, a sliding-window analysis for each pair of duplicated *ZmCHS* genes was also carried out in order to obtain the *K*a/*K*s ratios at different sites of coding sequences. The results suggested that *K*a/*K*s ratios of the *CHS* conserved domains including Chal_sti_synt_C domain and Chal_sti_synt_N domain were <1, suggesting negative selection effect (Table 3, Figure 5). We speculated that purifying selection might have contributed for the maintenance of function in maize *CHS* family to some degree.

**Table 3:** *K*a/*K*s analysis for the duplicated *ZmCHS* paralogs.

Duplicated Pairs	*K*a	*K*s	*K*a/*K*s	Purifying Selection
*ZmCHS01-ZmCHS02*	0.015	0.155	0.095	Yes
*ZmCHS13-ZmCHS14*	0.027	0.22	0.123	Yes

### 2.5. Microarray Analysis of CHS Expression during Maize Development

**Figure 6:**
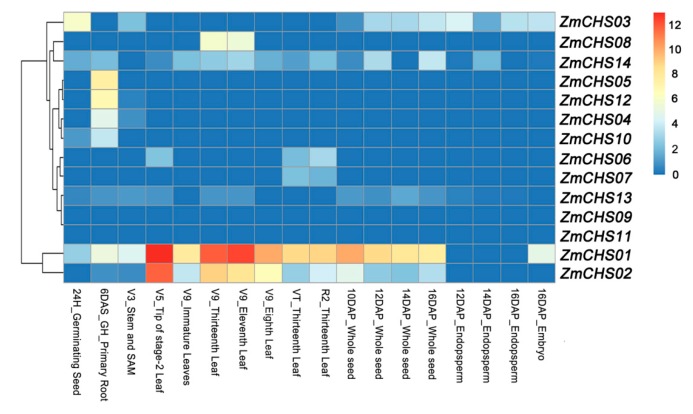
Clustering of expression profiles of 14 *ZmCHS* genes. Different organs/tissues are exhibited below each column. 24H, 24 h after imbibition; DAS, days after sowing; GH, growth hormone; SAM, Shoot apical meristem; V3, Vegetative 3, three fully extended leaves; V5, Vegetative 5, five fully extended leaves; VT, Vegetative tasseling, last branch of the tassel fully emerged; R2, Reproductive 2, 10–14 days after silk emergence; DAP, days after pollination. The names of *ZmCHS* genes are displayed at the right side of each row. The colour box from blue to orange indicate an increased expression level.

The expression patterns of genes are usually related to their function. To obtain the expression patterns of maize *CHS* genes, the maize microarray data by Sekhon *et al.* [35] were downloaded and analysed. The expression patterns of the 14 *ZmCHS* genes in diverse organs at different developmental stages were investigated by cluster expression profiles (Figure 6). The results revealed that the expression patterns of *CHS* genes were diverse. The majority of the *CHS* gene family members showed little expression in most maize tissues, including seeds, leaves, endosperm and embryo. The exceptions to this were *ZmCHS01/02*, which showed elevated levels of constitutive expression in seeds and all stages of leaves development (Figure 6).

### 2.6. Expression Levels of Maize CHS Genes in Response to Salicylic Acid Treatment

**Figure 7:**
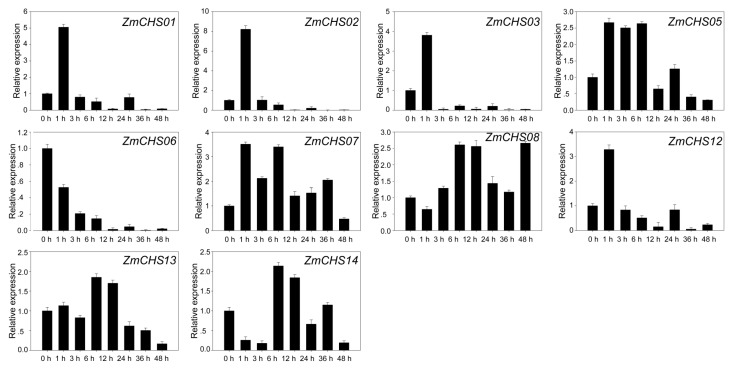
Expression patterns of 10 *ZmCHS* genes after salicylic acid treatment. Relative expression levels of *ZmCHS* genes in response to salicylic acid were examined by qPCR and normalized by the expression of Maize Actin 1 (NM_001136991.1). The *y*-axis represents the relative expression level and the *x*-axis represents the time course of stress treatment. Seedlings were sampled at 0, 1, 3, 6, 12, 24, 36 and 48 h after salicylic acid treatment. There were three technical replicates for each of the three biological replicates.

Expression of the *CHS* genes can be stimulated by various biotic and abiotic elicitors including light, infection, mechanical wounding and plant hormones [36,37,38]. Therefore, we examined the expression levels of the *ZmCHS*s in response to abiotic stress by subjecting maize seedlings to salicylic acid. No expression was detected for four genes (*ZmCHS*04, -09, -10, -11); therefore, the remaining 10 maize *CHS* genes were chosen for further qPCR analysis. The result revealed diverse expression patterns following salicylic acid treatment. As shown in Figure 7, the expression levels of the 10 *ZmCHS*s were compared to untreated plants and were induced or repressed by this stress treatment, albeit some induction was slight. After salicylic acid treatment, the expressions of *ZmCHS01*, *-02*, *-03*, *-05*, *-07* and *-12* were highly induced at a relatively early stage (1 h after treatment), while expressions of *ZmCHS13* and *-14* peaked at 6 h after treatment. We noted that the expression of *ZmCHS06* was down-regulated following salicylic acid treatment at all time points. Interestingly, the expression patterns of the segmental duplicated genes (*ZmCHS01/02*) displayed high similarity after salicylic acid treatment. Analogously, the expression patterns of the other segmental duplicated genes (*ZmCHS13/14*) exhibited similarity and both peaked at 6 h after treatment.

## 3. Discussion

The *CHS* gene family plays important roles in the growth and development of plants. Most plant genomes contain smaller *CHS* gene families. For example, in *Petunia hybrida*, eight complete *CHS* genes and four partial genes have been cloned and sequenced [39,40]. Six *CHS* genes were identified in *Ipomoea* [41]. At least eight *CHS* members were identified in pea (*Pisum sativum*) [42]. In this study, 14 *CHS* genes were identified in maize, which is one of the largest numbers of *CHS* family members identified in one organism to date, and is approximately consistent with previously reported numbers (15 or more) [25]. It is considered that gene duplications have led to the wide expansion of the gene family during evolutionary process. The genome of maize has gone through several rounds of duplications early in its evolutionary progress [43,44]. Two segmental duplication events were found among the 14 *ZmCHS* genes. Thus, around 29% of *ZmCHS* genes arose from the duplicated chromosomal regions. In contrast, no tandemly duplicated genes were found. These results suggested that segmental duplication was the dominating contributor for the expansion of maize *CHS* family. It was previously reported that there were 13 members encoding active CHS or CHS-like enzymes in physcomitrella patens *CHS* family [28], indicating that the ancient ancestors of plants already contained many *CHS* genes. So we speculated that a part of the 10 non-replicated *ZmCHS* genes might have arisen from the *CHS* genes of the ancestor moss. In addition, two maize *CHS* genes (*C2* and *Whp*), and one anther specific chalcone synthase-like (*ASCL*) gene (*ZmCHSL*, NP_001149508) were previously reported [22,23,24]. Analysis of the sequence alignment between the 14 genes and *C2*, *Whp* and *ZmCHSL* (NP_001149508) were conducted, and the high identities allowed us to conclude that *ZmCHS01* is *C2, and ZmCHS02* and *ZmCHS11* are likely to be *Whp* and *ZmCHSL*, respectively.

All *ZmCHS* genes could be broadly classified into four major classes: Classes I, II, III and IV based on their phylogenetic relationships. The motif distribution by Multiple Expectation Maximization for Motif Elicitation (MEME) was basically consistent with the phylogenetic analysis. The members in the same subfamily usually shared subfamily-specific conserved motifs. Furthermore, the two domains Chal_sti_synt_C and Chal_sti_synt_N, defined by motif distribution, were highly conserved in almost all of the *ZmCHS* genes, suggesting conserved evolution. Motifs 1 and 4 include the catalytic triad of CHN and are considered to be very important for the catalytic function. The catalytic triad inherited from the KAS III ancestor [3] are highly conserved in all of the ZmCHSs. There are some substitutes at positions Phe215 and Phe265, which are connected with CoA-binding [3]. These substitutions probably resulted in the different choice of the substrate. All the ZmCHSs contain the CHS family-specific Pro375 [3] (Figure 3), indicating the conserved evolution. Furthermore, the sequence alignment of the putative maize CHS proteins with MsCHS and other plant CHSs exhibited high similarity in sequences, suggesting that the CHS family are conserved in different species.

The gene structure analysis not only supported the phylogenetic analysis, but also revealed that the *CHS* genes were highly conserved during the evolutionary process of maize. In this study, most of the *CHS* genes (12 of 14) contained two exons and one intron, which was consistent with the previously proposed structure comprising two exons and one intron [45]. The gene duplication events also implied the conserved evolution of maize *CHS* genes. Previous studies suggested that the genome of maize has gone through several rounds of duplication. The duplication included an ancient genome duplication about 50–70 million years ago (Mya) when the grass genomes had not yet diverged, leading to the ancient tetraploid ancestry, another whole genome duplication approximately five Mya after the divergence of sorghum and maize and a recent duplication event [29,46,47,48]. In general, tandem duplications contribute to generate new genes [49]. However, segmental duplications tend to disperse gene copies [49], leading to them evolving slowly [33,50]. In this study, the presence of segmental duplications among the *CHS* genes indicates that this gene family is conserved and slowly evolving in maize.

Gene expression patterns are an important aspect of the study of gene function, and genes with similar expression patterns may have common features, be regulated by the same gene, or possess a common origin [51,52]. High-throughput microarray technology provides a good platform for the study of genome-wide gene expression patterns. The transcription profiles of maize revealed that the gene expression levels were highly diverse among tissues, with a variation ranging from 10% to 744% [35]. In this study, the majority of *ZmCHS* gene family members showed little expression in most maize tissues, except for *ZmCHS01/02*. Most *ZmCHS* genes exhibited variable expression patterns, suggesting functional diversification of *ZmCHS* genes. In addition, along with multiple functions of CHSs, the expression of these genes might occur under specific environments or is specific to an organ or developmental stage. For example, the expression of certain *CHS* genes in plants is affected by methyl jasmonate (MeJA), abscisic acid (ABA), salicylic acid (SA) [30] and light [28]. Moreover, many genes exhibited similar expression patterns, which might in turn catalyze similar substrates in the same biochemical pathway. In conclusion, the expression profiling in this study provides an important basis for further studying of expression and biological functions of the *CHS* gene family in maize.

The growth and development of plants is usually threatened by stresses from surroundings, such as drought, low temperature and high salinity during their life cycles. A lot of stress-related genes were induced to adapt to these environmental stresses [53,54]. The *CHS* family has been demonstrated to be regulated by salicylic acid treatment [30]. However, no *CHS* genes responding to salicylic acid stress have been reported in maize. Thus, we performed a survey of the expression patterns of the *CHS* genes in maize under salicylic acid stress. In this study, four *CHS* genes (*ZmCHS*04, -09, -10, -11) showed no detectable expression, which was also clearly shown in the heat map. The expression patterns of the other 10 maize *CHS* genes were investigated under salicylic acid treatments. The results suggested that all ten genes were responsive to salicylic acid treatment. More than half of the genes were highly expressed at a relatively early stage (1 h after treatment), and showed similar expression patterns. This indicated that these genes might execute their functions simultaneously. By contrast, the expression levels of a small number of genes peaked at six h after treatment, indicating that these genes are involved in a late-stage response to salicylic acid. In addition, the down-regulated gene *ZmCHS06* might also have specific functions in maize under salicylic acid treatment, such as defense [55]. We hypothesized that most *CHS* genes play essential roles in response to abiotic stresses such as salicylic acid. This conclusion was supported by the close relationship of *ZmCHS* to *PaCHS*, whose expression was also increased after salicylic acid treatment [30]. These results indicated that most *CHS* genes were induced by salicylic acid and might contribute to the defense against abiotic stresses or disease resistance [55,56,57] in maize. We noted that the two pairs of duplicated genes, *ZmCHS01/02* and *ZmCHS13/14* had very similar expression patterns in response to salicylic acid treatment. This indicated that the duplicated genes might have redundant functions in response to the abiotic stress. In addition, the *K*a/*K*s ratios of the two segmental duplication pairs were <1, indicating purifying selection, which confirmed our conclusion that the duplicated *CHS* genes mainly went through narrow functional divergence after duplication events.

In this study, we report our findings on the gene architecture, evolution and expression response to salicylic acid of the *CHS* genes in maize. In conclusion, the conservation and diversity of the *CHS* family genes make maize a model system for further studying of the functions, regulation and evolution of the *CHS* family.

## 4. Materials and Methods

### 4.1. Identification and Sequence Analysis of CHS Proteins in Maize

Firstly, we downloaded the complete gene sequences and protein sequences of maize from the database [58], and built a local database using the DNATOOLS software (Savita Shanker, FL, USA). We obtained the conserved domain sequences of the CHS family using Hidden Markov Model (HMM) while searching the Pfam [31]. Subsequently, this conserved domain sequences were employed to search for all CHS proteins in the local protein database using the BlastP program (Savita Shanker, FL, USA) (*p*-value = 0.001). Pfam and SMART [59] were then used to authenticate each CHS protein, which was key for the identification of the valid CHS proteins. We aligned all the candidate CHS sequences using ClustalW software (Kyoto University Bioinformatics Center, Kyoto, Japan) [60] and excluded potential redundant sequences. Finally the non-redundant *CHS* genes were obtained for further analysis. In addition, we calculated the molecular mass (Da) and isoelectric point (pI) of every protein using ExPASy (SIB Swiss Institute of Bioinformatics, Lausanne, Switzerland).

### 4.2. Phylogenetic Analysis

Protein sequence alignment was performed using the Clustal X program (Des Higgins, DUB, Ireland). Then, phylogenetic tree was built using the NJ (neighbor-joining) method with bootstrap 1000 using MEGA 4.0 (Koichiro Tamura, TKY, Japan [61]. According to the phylogenetic relationships, we classified the *ZmCHS* genes into subfamilies.

### 4.3. Analysis of Conserved Motifs, Gene Structure and Sequence Alignment 

We investigated the conserved motifs in each putative ZmCHS protein using the MEME web sever [62]. The following parameters were set: width of motif is ≥6 and ≤200 and maximum number of motifs to find is 20. In addition, the gene structure analysis, including exon and intron, was performed by comparing the gene sequences with the predicted coding sequences using Gene Structure Display Server (GSDS) [63].

The crystal structure of alfalfa CHS [64] has revealed the configuration of amino acid residues in many active sites. Studies on the structure and function of the MsCHS protein have been thorough and comprehensive. So we used the secondary structure of MsCHS as a template to analyze the secondary structures of maize CHSs using the online server Protein Data Bank (PDB) [65] and ESpript [66]. An alignment of alfalfa CHS, ZmCHSs and other plant CHS proteins was performed using the ClustalW software (Kyoto University Bioinformatics Center, Kyoto, Japan).

### 4.4. Chromosomal Locations and Gene Duplication

The physical map of the *ZmCHS* genes was drawn by using MapInspect [67], according to their positions on the chromosomes of maize. The MCScan software [68] was used to identify the duplicated *ZmCHS* genes. The maize whole-genome sequences were downloaded to our local server and were analyzed using an all-versus-all Basic Local Alignment Search Tool (BLAST) search with an *E*-value less than 1 × 10^−5^. Subsequently, the MCScan software was used to analyze the synteny regions [68]. The pairs of genes located in these duplicated regions were considered as segmental duplication gene pairs and were indicated on the physical map.

### 4.5. Microarray Analysis of CHSs in Maize

Publically available transcriptome data by Sekhon *et al.* [35] were obtained to examine the expression patterns of *ZmCHS* genes. The gene expression patterns in roots, seeds, leaves, endosperm and embryo were drawn using R/Bioconductor [69].

### 4.6. Plant Material and Salicylic Acid Stress Treatment

To measure the expression levels of *CHS* genes under abiotic stress, the seeds of maize inbred line B73 were grown in the greenhouse at 28 ± 2 °C with a 14 h light/10 h dark photoperiod. After three weeks, the leaves of the maize (five-leaf stage seedlings) were sprayed with 100 mM salicylic acid solution and sampled at 0, 1, 3, 6, 12, 24, 36 and 48 h after treatment. There are three biological replicates for each sample.

### 4.7. RNA Isolation and Quantitative Real-Time PCR (qPCR) Analysis

The leaves of the maize seedlings sprayed with salicylic acid solution were sampled at 0, 1, 3, 6, 12, 24, 36 and 48 h after treatment for RNA isolation. Total RNA was extracted according to the CTAB method [70]. Then DNaseI (Invitrogen, Shanghai, China) was used to remove residual DNA, followed by reverse-transcription using Moloney Murine Leukemia Virus (M-MLV) reverse transcriptase (Invitrogen, Shanghai, China). The ABI 7300 Real-Time system (Applied Biosystems, Foster, CA, USA) was used to perform the qPCR (quantitative real-time PCR). Primer Express 3.0 software (Applied Biosystems, Foster, CA, USA) was used to design the specific primers for amplifying each *ZmCHS* gene (Table S1). Each reaction contained a system of 20 µL including 12.5 µL of SYBR Green Master Mix reagent (Applied Biosystems), 400 nM specific primers and 1.5 µL of cDNA. The qPCR reaction conditions were set as follows: 95 °C for 10 min; followed by 40 cycles at 95 °C for 15 s and 60 °C for 1 min. Subsequently, a melting curve was formed to check the specificity of the genes. The qPCR was carried out with three technical replicates for each of the three biological replicates. The expression level of the maize Actin 1 gene was treated as an internal reference. The relative expression level of each gene was calculated as 2 ^−∆∆*C*t^ [∆*C*_t_ = *C*_t_,_Target_ − *C*_t_,_Actin 1_; ∆∆*C*_t_ = ∆*C*_t_,_treatment_ − ∆*C*_t_,_CK (0h)_] [71].

## Supplementary Materials

Supplementary materials can be found at http://www.mdpi.com/1422-0067/17/2/161/s1.
