# Supplementary material for: Genome-Wide Identification, Characterization and Expression Analysis of the Chalcone Synthase Family in Maize
Source: Int J Mol Sci. 2016 Jan 27;17(2):161. doi: 10.3390/ijms17020161 (PMC4783895; doi:10.3390/ijms17020161)
Supplement: Supplementary file 1 [file ijms-17-00161-s001.pdf]

Supplementary Materials: Genome-Wide Identification, Characterization and Expression Analysis of the Chalcone Synthase Family in Maize

Yahui Han, Ting Ding, Bo Su and Haiyang Jiang

Table S1. The specific-primers for qPCR.

ID	Gene Name	Primer 5′→3′
1	*ZmCHS01-F*	AGCACCTCACCGACCTCAAG
2	*ZmCHS01-R*	ACGCTTCCGGATCATCGA
3	*ZmCHS02-F*	TCACCGACCTCAAGGAGAAGTT
4	*ZmCHS02-R*	TGTACCGCTTCCGGATCATC
5	*ZmCHS03-F*	CTGGCCTCAAGGAAAAGTTCAA
6	*ZmCHS03-R*	CCTCGGTGTGGTGCAGGTA
7	*ZmCHS04-F*	GCGAGCACCTCACTAGCCTTA
8	*ZmCHS04-R*	GTGGTGGAAGTAGCGTTTCTTGA
9	*ZmCHS05-F*	AGAACCGGACTTCGAGATGCT
10	*ZmCHS05-R*	CGGCGACCTGCTTGCT
11	*ZmCHS06-F*	GCTGAGAGAGTACGGCAATATGAG
12	*ZmCHS06-R*	CGTCAGTCGGCGGTGAAC
13	*ZmCHS07-F*	CGTCGTCCCGCCCATT
14	*ZmCHS07-R*	CTGAGCAGCGGATCCTGTTAA
15	*ZmCHS08-F*	GCCGCCGTGAGGAAGAC
16	*ZmCHS08-R*	GGCAGCATGCAGCACAATT
17	*ZmCHS09-F*	CTGCGTTCAGCAAGACGAGTAC
18	*ZmCHS09-R*	AGGTGGTCGCTGTTGGTGATA
19	*ZmCHS10-F*	ATCATCTTACACACACGAAAGCAATC
20	*ZmCHS10-R*	GACTTGCAGGTATCGTTTTTTTACC
21	*ZmCHS11-F*	GAAGTAAGTTGCTTTGCGATGGT
22	*ZmCHS11-R*	GGCCTTGCCAGGGTTAGG
23	*ZmCHS12-F*	CCGTCGAAGAACCGGACTT
24	*ZmCHS12-R*	GTGCAACCCATCCTCACAGA
25	*ZmCHS13-F*	CACAACAGTACAGCACTGGAAACA
26	*ZmCHS13-R*	GCTGCCAGTGTCCGTCATT
27	*ZmCHS14-F*	TATAGGAGCAGGTCCCATGACA
28	*ZmCHS14-R*	GGAACTCCTGCGTGGAGAAC
